# Songbirds initiate migratory flights synchronously relative to civil dusk

**DOI:** 10.1186/s40462-023-00382-5

**Published:** 2023-05-01

**Authors:** Nathan W. Cooper, Bryant C. Dossman, Lucas E. Berrigan, J. Morgan Brown, Alicia R. Brunner, Helen E. Chmura, Dominic A. Cormier, Camille Bégin-Marchand, Amanda D. Rodewald, Philip D. Taylor, Christopher M. Tonra, Junior A. Tremblay, Peter P. Marra

**Affiliations:** 1 Migratory Bird Center, Smithsonian’s National Zoo and Conservation Biology Institute, 3001 Connecticut Ave. NW - MRC 5503, Washington, DC 20008 USA; 2 Department of Biology and McCourt School of Public Policy, Georgetown University, 37th and O Streets NW, Washington, DC 20057 USA; 3 Cornell Lab of Ornithology, Department of Natural Resources and the Environment, Cornell University, 159 Sapsucker Woods Rd, Ithaca, NY 14850 USA; 4 Department of Biology, Acadia University, 33 Westwood Avenue, Wolfville, NS B4P 2R6 Canada; 5 Motus Wildlife Tracking System, N0E 1M0 Birds, Port Rowan, ON Canada; 6 Institute for Biodiversity and Ecosystem Dynamics, University of Amsterdam, 904 Science Park, 1098XH Amsterdam, The Netherlands; 7 School of Environment and Natural Resources, The Ohio State University, 2021 Coffey Rd, 43210 Columbus, OH USA; 8 Rocky Mountain Research Station, USDA Forest Service, 800 East Beckwith Avenue, 59801 Missoula, MT USA; 9 Wildlife Research Division, Environment and Climate Change Canada, 1550 Av. D’Estimauville, G1J 0C3 Québec, QC Canada

**Keywords:** Biological timing, Celestial cues, Circadian rhythm, Civil dusk, Navigation, Nocturnal departure timing, Migration phenology, Orientation, Sunset

## Abstract

**Background:**

Each spring and fall billions of songbirds depart on nocturnal migrations across the globe. Theory suggests that songbirds should depart on migration shortly after sunset to maximize their potential for nightly flight duration or to time departure with the emergence of celestial cues needed for orientation and navigation. Although captive studies have found that songbirds depart during a narrow window of time after sunset, observational studies have found that wild birds depart later and more asynchronously relative to sunset than predicted.

**Methods:**

We used coded radio tags and automated radio-telemetry to estimate the time that nearly 400 individuals from nine songbird species departed their breeding or wintering grounds across North America. We also assessed whether each species was most likely beginning long-distance migratory flights at departure or instead first making non-migratory regional flights. We then explored variation in nocturnal departure time by post-departure movement type, species, age, sex, and season.

**Results:**

We found that 90% of individuals from species that were likely initiating long-distance migratory flights departed within 69 min of civil dusk, regardless of species, season, age, or sex. By contrast, species that likely first made non-migratory regional movements away from the migratory destination departed later and more asynchronously throughout the night. Regardless of post-departure movement type, 98% of individuals departed after civil dusk but otherwise showed no preference in relation to twilight phase.

**Conclusions:**

Although the presence of celestial orientation cues at civil dusk may set a starting point for departure each night, the fact that species likely beginning long-distance migration departed earlier and more synchronously relative to civil dusk than those first making non-migratory regional movements is consistent with the hypothesis that departing promptly after civil dusk functions to maximize the potential for nightly flight duration and distance. By studying the onset of migration, our study provides baseline information about departure decisions that may enhance our understanding of departure timing throughout migration.

**Supplementary Information:**

The online version contains supplementary material available at 10.1186/s40462-023-00382-5.

## Background

To synchronize their physiology and behavior with the annual and diel rhythms of the environment, organisms have evolved endogenous time-keeping mechanisms such as circannual and circadian clocks [1, 2]. Organisms use these clocks to time both critical transitions within the annual cycle (e.g., migration, hibernation, reproduction) and important daily routines (e.g., sleep-wake cycles, foraging, locomotion). Despite endogenous control, the realized timing of these behaviors in the wild is often plastic, which allows individuals to adaptatively respond to important social and environmental factors [1, 3, 4]. Long-distance migratory songbirds, for instance, use circannual clocks that are synchronized by photoperiod to time their seasonal migrations [5], but their realized migration phenology is influenced by diverse factors including social dominance hierarchies, habitat quality, food availability, and the timing of recent reproductive efforts [6–9]. The diel timing of avian migration is also under endogenous control, by a circadian clock that is synchronized by the light-dark cycle [10–15]. Captive songbirds demonstrate tight endogenous control of the diel timing of migration by initiating migratory behavior during a narrow window of time after sunset [10, 14, 15], but observational studies on wild songbirds have documented more variable departure times [16]. Understanding this discrepancy between captive-based studies and free-living individuals is key to understanding the ecological and environmental underpinnings of the diel timing of migration.

Most songbirds migrate nocturnally [17], and by flying at night songbirds increase the time they have available for daytime foraging and reduce their energetic costs, risk of dehydration, exposure to turbulent winds, and predation risk while in flight [18, 19]. Although individuals could realize these benefits by initiating migratory flights at any time during the night, two non-mutually exclusive hypotheses predict that songbirds should begin migratory flights shortly after sunset. The duration-maximization hypothesis argues that songbirds should depart on migration shortly after sunset to maximize the amount of time they have available to fly each night [16]. Nightly flight duration and flight speed combine to determine distance flown and maximizing nightly flight duration could therefore be one aspect of a migration strategy that functions to ensure timely arrival to their wintering and breeding destinations. The celestial cues hypothesis [20, 21] also suggests that individuals should depart shortly after sunset, but predicts that the exact time of departure will depend on the availability of celestial cues (i.e., sunset position, skylight polarization patterns, and stars), which are needed for avian orientation and navigation and gradually appear and disappear during evening twilight [22–24].

Despite long-held predictions that songbirds should initiate migratory flights during a narrow window of time after sunset and their demonstrated ability to do so in captivity, observations of individual birds in the wild have repeatedly failed to match theory or captive studies. The general pattern observed in wild songbirds thus far is that although many individuals depart within the first four hours after sunset, departure can occur at any time between sunset and sunrise [16, 20, 21, 25–34]. Many factors have been hypothesized to account for variation in nocturnal departure time including age, sex, fuel load, parasite infection, the presence of ecological barriers, distance remaining to the migratory destination, and the type of movement initiated at departure [16, 29, 31, 35, 36]. Nonetheless, why nocturnal departure times observed in the wild have been more variable than in captive studies or predicted by theory remains unclear.

One clue may be that nearly all field studies of nocturnal departure time have been conducted at stopover sites, rather than at breeding or wintering sites, when birds are first initiating migration for the season (but see [37]). Stopover sites serve a variety of functions in birds including refueling, recovering from physiological stress, escaping predators, and avoiding inclement weather [38, 39]. As a result, individuals may vary in their energetic state upon arrival, duration of stay at the stopover site, and immediate goals after departing. Upon departure, individuals may either recommence migration or relocate to another nearby stopover site through non-migratory regional movements, presumably to find better quality habitat (i.e., habitat with more food, fewer predators, etc.) [31, 40]. Accordingly, individuals departing stopover sites likely vary in their need to maximize flight duration or observe celestial orientation cues and may subsequently depart at variable times of night [29, 31, 41]. Even among those birds continuing long-distance migration, individuals may show large amounts of variation in fuel load [39]. Moreover, because of potentially highly variable breeding or wintering destinations, their migratory route and proximity to ecological barriers the night after departure, and distance to the next destination may vary considerably, all of which could affect their nocturnal departure time [16]. Compared to individuals at temporary stopover sites, birds departing their long-term wintering and breeding sites have typically had much more time to increase fuel loads, and because they are just beginning their migrations likely vary considerably less in terms of immediate differences in migratory route, proximity to ecological barriers, and distance remaining on migration. Controlling for these sources of variation by studying departure from wintering and breeding sites could reveal new insights into nocturnal departure time and help explain the discrepancy between theory, captive experiments, and previous observational studies.

We used automated radio telemetry [42] to estimate nocturnal departure time in nine species of songbirds departing six breeding or wintering sites across North America (Table 1, Table S1). Most species in our study are thought to begin long-distance migration after departure from their breeding or wintering sites (see Methods), but three species are known to depart their breeding sites and primarily carry out non-migratory regional movements for days or weeks before beginning directed long-distance flights towards their wintering sites [43–46]. Because of this apparent variation in post-departure movement strategy, we used radio-tracking data from outside of the breeding and wintering sites to assess whether species were most likely initiating long-distance migratory flights after departure or instead first making non-migratory regional movements. We then used automated telemetry data from our breeding and wintering sites to: (1) document nocturnal departure time, (2) determine whether species-level differences in post-departure movement type (i.e., migratory or regional flights) resulted in variation in nocturnal departure time, (3) explain variation in nocturnal departure time based on age, sex, season, and species, and (4) determine if cloud cover and its effects on the visibility of celestial orientation cues led to differences in departure time. Finally, we explored what the observed patterns of nocturnal departure time reveal about the functional significance of this behavior and its implications for our understanding of avian orientation and navigation.

**Table 1:** Post-departure movement type, sample size, departure location, and migratory destination are shown for each species included in the study. See references for habitat and tagging information. See also Table S1 for more details

Species	Movement type	*n* (sex)	Departure location	Destination	References
American Redstart (Spring) *Setophaga ruticilla*	Migration	31 (12♀, 19♂)	Jamaica	Great Lakes Region, N.E. USA	[6]
Ovenbird (Spring) *Seiurus aurocapilla*	Migration	17 (sex unk.)	Jamaica	N.E. USA	Brunner et al. unpub. data
Swainson’s Warbler (Spring) *Limnothlypis swainsonii*	Migration	14 (sex unk.)	Jamaica	S.E. USA	[78]
Kirtland’s Warbler (Spring) *Setophaga kirtlandii*	Migration	66 (13♀, 53♂)	The Bahamas	Great Lakes Region	[79]
Kirtland’s Warbler (Fall) *Setophaga kirtlandii*	Migration	46 (11♀, 35♂)	Michigan	The Bahamas	[79]
Gambel’s White-crowned Sparrow (Fall) *Zonotrichia leucophrys gambelii*	Unk.	49 (21♀, 28♂)	Alaska	Western USA	[9]
Bicknell’s Thrush (Fall) *Catharus bicknelli*	Migration	19 (17♂, 2 unk.)	Quebec	Hispaniola and Cuba	[46]
Swainson’s Thrush (Fall) *Catharus ustulatus*	Unk.	23 (5♀, 15♂, 3 unk.)	Quebec	Central and South America	[46]
Swainson’s Thrush (Fall) *Catharus ustulatus*	Regional	65 (2♀, 14♂, 49 unk.)	Nova Scotia	Central and South America	[45]
Blackpoll Warbler (Fall) *Setophaga striata*	Regional	49 (9♀, 5♂, 35 unk.)	Nova Scotia	South America	[43, 44]
Yellow-rumped Warbler (Fall) *Setophaga coronata*	Regional	17 (5♀, 3♂, 9 unk.)	Nova Scotia	S. USA, Caribbean, C. America	[44]

## Methods

To enhance our ability to draw broad conclusions, we collected data from nine species of migratory songbirds from two wintering sites and four breeding sites in North America (Table 1, Table S1). All species are long-distance migratory songbirds with the possible exception of Yellow-rumped Warblers (*Setophaga coronata*), which may migrate medium distances to the mid-Atlantic or long distances to Central America or the Caribbean (Table 1). For one species, Kirtland’s Warblers (*Setophaga kirtlandii*), we collected data from the same population during both spring and fall migration, and for another, Swainson’s Thrush (*Catharus ustulatus*), we collected data from the same season, but at two different locations (inland Quebec and coastal Nova Scotia, Canada). All individuals were captured in mist nests, aged and sexed when possible [47], and banded with one aluminum U.S. Geological Survey band and up to three colored plastic bands. We then attached a radio tag to each bird using a modified leg-loop harness [48]. Radio tag model, weight, and pulse rate varied by species (Table S1).

### Post-departure movement type

Previous light-level geolocator and/or radio-tracking data from the same breeding and wintering sites as the present study provides no clear evidence that Kirtland’s Warblers, American Redstarts (*Setophaga ruticilla*), Ovenbirds (*Seiurus aurocapilla*), Swainson’s Warblers (*Limnothlypis swainsonii*), or Quebec-breeding Swainson’s Thrushes make extensive non-migratory regional movements after departure [46, 49–52]. By contrast, Swainson’s Thrushes, Blackpoll Warblers (*Setophaga striata*), and Yellow-rumped Warblers breeding at the same Nova Scotia breeding sites as the present study have all been previously documented making non-migratory regional movements for days or weeks after departure [43–46]. Previous tracking data are unavailable for Bicknell’s Thrush (*Catharus bicknelli*) or Gambel’s White-crowned Sparrows (*Zonotrichia leucophrys gambelii*). To account for this apparent difference in post-departure movement type between species within our sample, we tracked individuals after departure using the Motus Wildlife Tracking System [42].

**Fig. 1:**
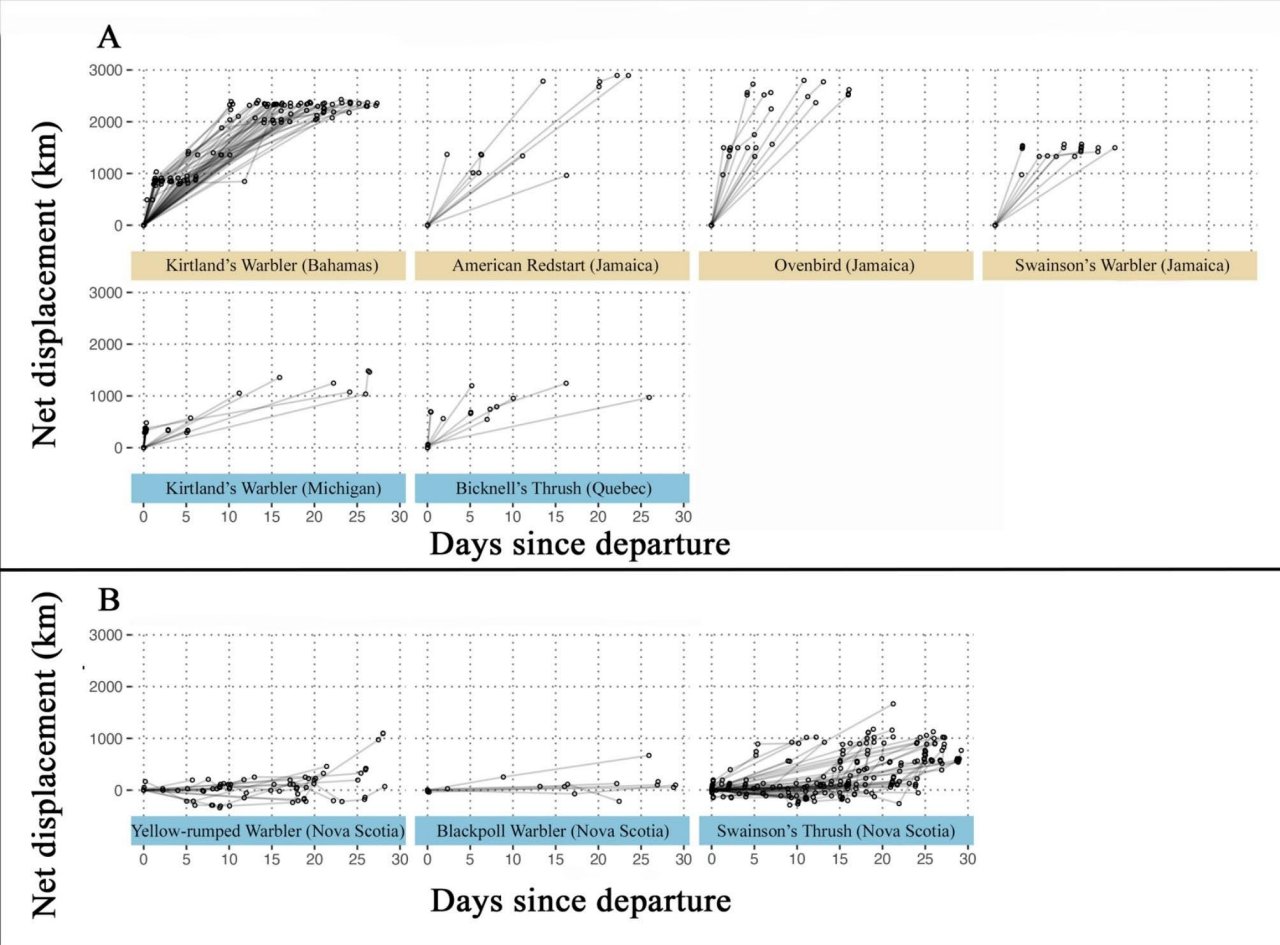
Net displacement (km) towards the migratory destination by time since departing wintering (beige) or breeding sites (blue) for species likely initiating long-distance migratory flights at departure (Panel A) and species likely first making regional flights after departure (Panel B). Quebec-breeding Swainson’s Thrush and Gambel’s White-crowned Sparrows are not shown because we did not have enough post-departure detection data to make conclusions about their likely post-departure movement type

The temporal and spatial resolution of automated telemetry data primarily depends on the movement of the animal relative to the spatial arrangement of automated telemetry radio stations (hereafter stations). Variation in the density and location of stations outside of the wintering and breeding areas for each species prevented us from definitively determining the distance travelled for each individual on the night of departure. To assess whether departing individuals were most likely initiating non-migratory regional flights or long-distance migratory flights, we estimated net displacement distance for individuals that were detected at least once after departure. We defined net displacement as the total distance moved in the general direction of the eventual migratory destination (i.e., the breeding or wintering region individuals were migrating towards) each time an individual was detected by a station. To calculate net displacement, we first determined the bearing and great circle distance from the site of departure to the station each individual was detected at using packages “swfscMisc”[53], “geosphere” [54], and “motus” [55] in Program R [56]. We could not assess post-departure movement type for each individual because not all individuals were detected after departure. Instead, by visually inspecting plots of net displacement over time (Fig. 1), we inferred the dominant pattern of movement (migratory vs. regional) shortly after departure for each species at each breeding or wintering site. During data exploration, movements in the opposite direction of the eventual migratory destination were only detected in the three populations breeding in Nova Scotia (i.e., Swainson’s Thrushes, Blackpoll Warblers, Yellow-rumped Warblers). In these populations, we assigned a negative value to net displacement distances for detections that were located to the north or east (0–90º) of the breeding site because all three species winter to the south or southwest of their breeding sites. No Gambel’s White-crowned Sparrows were detected outside of their Alaskan breeding site because they were tagged with transmitters that were not compatible with Motus stations, and therefore we could not assess whether they were immediately initiating migratory or regional flights.

### Estimating nocturnal departure time

At the Alaska breeding site, we used two automated telemetry receivers that were not part of Motus to record departure of Gambel’s White-crowned Sparrows. Time of departure was estimated as the time of last detection at the breeding site [9]. At all other sites, we used a local array of automated radio-telemetry stations that were operated as part of the Motus Wildlife Tracking System [42]. To determine nocturnal departure time, we inspected detection data from stations located at the breeding or wintering sites to look for identifiable signals of departure from the breeding or wintering site. As birds depart, a rapid increase in signal strength occurs when individuals gain elevation and become more detectable, followed by a gradual decrease in signal strength as individuals fly away from the station. We recorded the time that signal strength peaked as the departure time [31, 57]. All species in our study are nocturnal migrants, and we removed any birds that appeared to depart after astronomical dawn (i.e., the end of night; *n* = 20) or between sunrise and sunset (*n* = 18), assuming that departure during morning twilight or after sunrise was indicative of birds making short-distance local movements through normal daily activities and/or small home range shifts out of range of the stations but without leaving the breeding or wintering site [37]. For the breeding site in Alaska, we excluded birds that departed after civil dawn (*n* = 3) rather than after astronomical dawn, because the latter is never experienced during the departure period due to Alaska’s extreme northern latitude.

To calculate nocturnal departure time in relation to the time of local sunset, civil dusk, and sun elevation angle, we used the package “suncalc” [58] in Program R [56]. Twilight phases are defined by the angle of the sun relative to the horizon and are associated with specific astronomical events. During civil twilight (0–^−^6º), sunset position is easily observable, skylight polarization patterns are at maximum visibility, and a few of the brightest stars become visible. During nautical twilight (^−^6–^−^12º), the position of the sun is not visible, skylight polarization gradually dims from 50 to 0% brightness, and dozens of additional stars become visible. During astronomical twilight (^−^12–^−^18º), light from the sun is no longer visible and stars eventually reach their maximum brightness [20, 21, 59, 60]. Once the sun moves more than 18º below the horizon, true night begins. Due to Earth’s axial tilt, twilight duration increases with latitude, and at extreme latitudes the sun may not decline below the horizon far enough for a location to experience all twilight periods. For example, at the Alaska field site (69°N) civil dusk began 61–109 min after sunset during the departure period, but only 24 min after sunset in The Bahamas (25°N), and Alaska never experienced astronomical twilight or true night during the departure period.

### Statistical analysis

For comparison of nocturnal departure time between species, we qualitatively described obvious differences because the shape of departure time distributions varied widely, violating assumptions of parametric and non-parametric central tendency tests. To test for differences in the variability of nocturnal departure, we used Fligner-Killeen tests. To compare nocturnal departure between seasons in Kirtland’s Warblers and between locations in Swainson’s Thrush, we used Mann-Whitney-Wilcoxon tests. To explore age, sex, and year effects on the timing of nocturnal departure, we used ANOVA or Kruskal-Wallis tests. To estimate cloud cover during the hour surrounding civil dusk for each individual, we downloaded hourly weather data (31 km resolution) for each breeding or wintering site from the Copernicus Climate Change Service’s ERA5 reanalysis [61]. We then compared nocturnal departure time between individuals leaving on overcast nights (≥ 95% cloud cover) and those departing on mostly clear nights (≤ 25% cloud cover) using Mann-Whitney-Wilcoxon tests. We carried out similar tests comparing overcast nights to partly cloudy (≤ 50% cloud cover) and partly clear (≤ 33% cloud cover) nights and results were qualitatively similar, but we only report results from comparisons between overcast and mostly clear nights for simplicity. Throughout, we report the median ± inter-quartile range (IQR) and all tests were two-tailed. All figures were created using package “ggplot2” [62] for Program R [56].

## Results

### Post-departure movements

All individuals included in the analyses initiated departure flights nocturnally and permanently departed their breeding or wintering sites for the season. Tracking data indicated that wintering Kirtland’s Warblers and Ovenbirds typically travelled > 500 km towards the breeding grounds in the first few nights after departure. American Redstarts and Swainson’s Warblers were less frequently detected within the first few nights after departure, making it more difficult to assess progress towards the migratory destination immediately after departure. However, because they generally appeared to move at similar rates to Kirtland’s Warblers and Ovenbirds, and previous tracking data from the same study sites has not provided evidence of regional movements post-departure, we classified them as migrating at departure (Fig. 1A). Overall, net displacement after departure from the breeding grounds accumulated more slowly than in spring, consistent with the idea that birds migrate more slowly in the fall, but the observed patterns of net displacement varied among species. Most Kirtland’s Warblers travelled at least a few hundred kilometers towards The Bahamas within the first night of departure, and we therefore classified them as migrating, but individuals were often not detected later during their migrations. The majority of tracked Bicknell’s Thrush also travelled hundreds of kilometers south with the first five nights of departure, and we therefore classified them as migrating at departure. In contrast to the above species, but consistent with our previous research [43–45], we found that among species breeding in Nova Scotia, including Blackpoll Warblers, Yellow-rumped Warblers, and all but a few Swainson’s Thrushes, individuals first made non-migratory regional flights to the north or northeast, in the opposite direction of their wintering grounds. These regional flights continued in various directions around Nova Scotia before long-distance migratory movements appeared to begin more than 10 days after departing the breeding site (Fig. 1B). Most Swainson’s Thrushes breeding in Quebec were detected 35–60 km south of the breeding site within the first couple of hours after departure and one individual travelled at least 385 km within the first night. However, detections were too sparse in this population to make any strong conclusions about their post-departure movement type (Fig. 1A). Similarly, we could not determine whether departure by Gambel’s White-crowned Sparrow was associated with long-distance migration or regional flights because no detection data outside of the breeding site were available. We therefore excluded Swainson’s Thrush breeding in Quebec and Gambel’s White-crowned Sparrow from summary statistics and analyses below that explore differences in nocturnal departure time and sun elevation angle at departure by post-departure movement type.

### Nocturnal departure timing

**Fig. 2:**
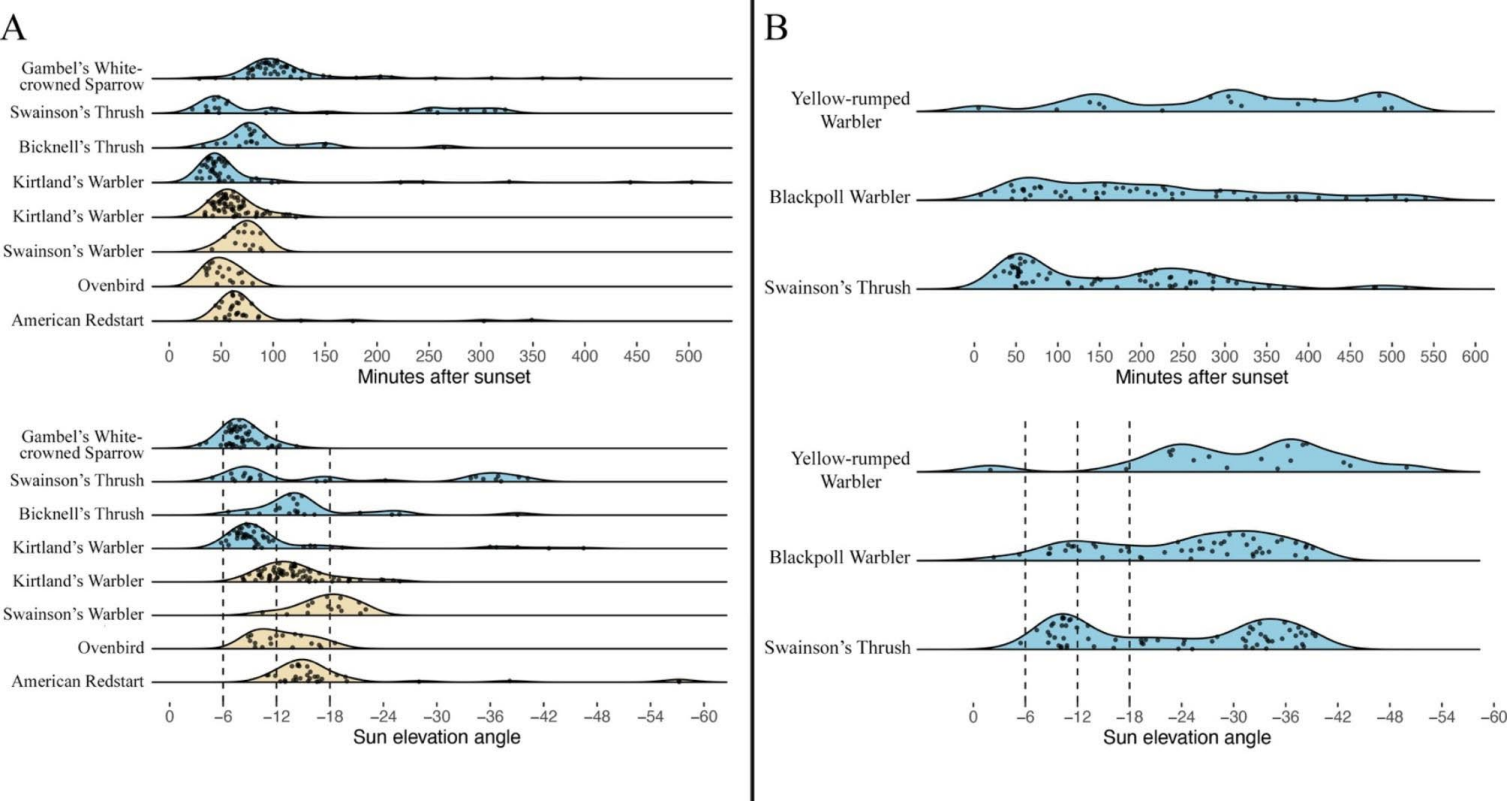
Nocturnal departure time from wintering (beige) and breeding sites (blue) in relation to sunset (top) and sun elevation angle (bottom) for species likely initiating long-distance migratory flights at departure (Panel A) and species likely first making regional flights after departure (Panel B). Gambel’s white-crowned sparrows are shown here with species initiating migratory flights because their distributions of departure times are similar, but we do not have post-departure detection data to confirm that they were beginning migration. Similarly, Quebec-breeding Swainson’s Thrush are shown with species likely beginning migration, but tracking data were too sparse to make conclusions about their post-departure movement type. Dashed lines indicate civil dusk (-6°), nautical dusk (-12°), and astronomical dusk (-18°). Note that sites in Alaska where Gambel’s white-crowned sparrows breed did not experience astronomical dusk during the departure period because of their northern latitude (see Methods)

The date of departure from breeding and wintering sites varied considerably, with individuals departing over a 13- to 43-day period depending on the species and season (Table S1). Across all species and locations, individuals departed on average 76.4 ± 98.7 (median ± IQR) minutes after sunset. Species that were likely beginning migration (Fig. 2A excluding Gambel’s White-crowned Sparrow and Quebec-breeding Swainson’s Thrush) departed two hours earlier on average and much more synchronously relative to sunset (60.4 ± 26.8 min) than those first making regional movements (Figs. 2B and 186.3 ± 219.3 min; Fligner-Killeen *X*^*2*^ = 130.7, *df* = 1, *n* = 324, *P* < 0.001). Similarly, species likely beginning migration departed at higher sun elevation angles and more synchronously with respect to sun angle (-13.8 ± 6.7º) than those first making regional movements (-26.1 ± 21.0º; Fligner-Killeen *X*^*2*^ = 66.1, *df* = 1, *n* = 324, *P* < 0.001; Fig. 2 bottom). Within species breeding in Nova Scotia, most individuals appeared to carry out non-migratory regional flights after departure (Fig. 1), but we identified four Swainson’s Thrush breeding in Nova Scotia that flew 391–670 km towards the wintering grounds within the first 9 days after departing and thus may have begun long-distance migration upon departure. These four individuals departed 45 to 70 min after sunset (-9.0 to -13.2º), well within the range of departure for species that were likely beginning migration, but earlier on average than conspecifics at the same site (147.6 ± 198.8 min after sunset; -23.6 ± 23.0º).

**Fig. 3:**
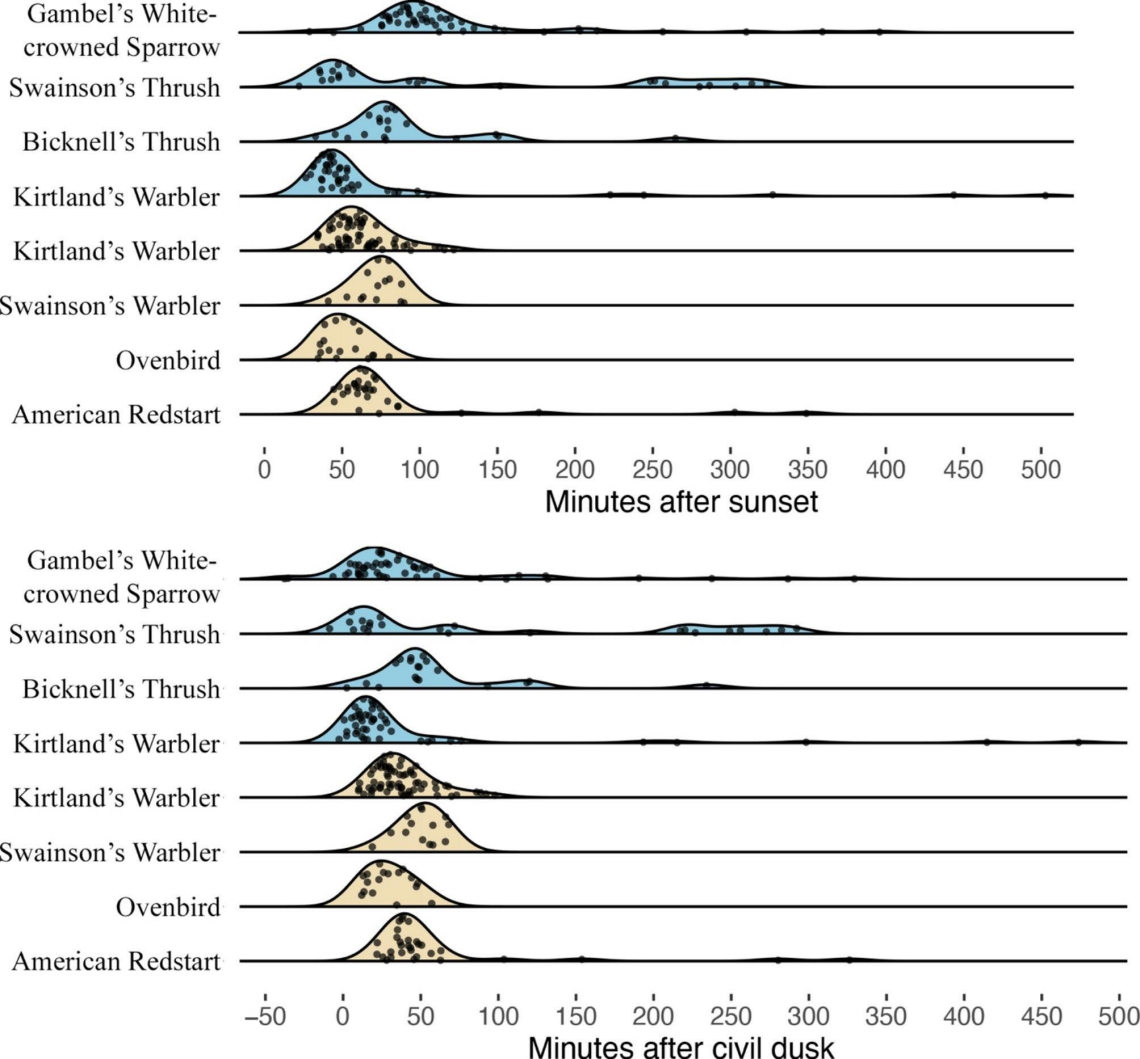
Nocturnal departure time from wintering (beige) and breeding sites (blue) in relation to sunset (top) and civil dusk (bottom) for species likely initiating long-distance migratory flights at departure. Notice the smaller difference between Gambel’s White-crowned Sparrow and all other species when using time after civil dusk compared to time after sunset. Quebec-breeding Swainson’s Thrush and Gambel’s White-crowned Sparrow are shown here with species likely initiating migratory flights because their distributions of departure times are similar, but we do not have post-departure detection data to confirm that they were beginning migration (see Methods)

Although individuals from species likely beginning migration primarily departed during a narrow window of time, this window of time spanned multiple periods of twilight both within and between species. Thus, regardless of post-departure movement type, we found no clear preference for departing during a single period of twilight (Fig. 2 bottom). This pattern was evident even when comparing the same species (Kirtland’s Warblers) departing in spring and fall. For a species to depart during a single period of time it would have required even less variable departure times than we observed because, at least outside of Alaska, the phases of twilight were of relatively short duration. We did notice however, that only 9 of the 396 (2.3%) individuals included in the study departed prior to civil dusk. We also noted that when comparing distributions of nocturnal departure using time after sunset and time after civil dusk, the variation between Gambel’s White-crowned Sparrow and species that were likely initiating migration was smaller when using time after civil dusk (Fig. 3). Accordingly, we found that Gambel’s White-crowned Sparrow departed significantly later after sunset (101.5 ± 40.2 min) than individuals from species that were likely beginning migration (60.4 ± 26.8 min; W = 8123, *n* = 242, P < 0.001), but this difference reversed and was no longer significant when using time after civil dusk (Gambel’s White-crowned Sparrow = 27.3 ± 39.1 min, all migrating species = 36.2 ± 27.7 min; W = 4144, *n* = 242, P = 0.182). As a result, we use time after civil dusk as a measure of departure time hereafter.

Within each species, we found almost no variation in nocturnal departure time by age, sex, or year, irrespective of the metric used (Tables 2 and 3). For American Redstarts, we initially found a significant year effect, but it was based entirely on the inclusion of two males that departed 280 and 326 min after civil dusk in 2019, much later than all other conspecifics (42.0 ± 12.4 min; Range = 22–154 min). When comparing Kirtland’s Warbler departure between seasons, we found that individuals leaving their Bahamian wintering grounds departed slightly later after civil dusk (35.7 ± 20.7 min) than individuals departing breeding areas in Michigan (18.5 ± 17.1 min; W = 842, *n* = 112, *P* < 0.001). When comparing Swainson’s Thrush from two different breeding locations, we found that individuals breeding in Quebec (62.4 ± 208.7 min) departed earlier after civil dusk than individuals breeding in Nova Scotia Swainson’s Thrush (117.7 ± 198.8 min), but the difference was not significant (W = 600, *n* = 88, *P* = 0.163).

**Table 2:** Age, sex, and year differences in nocturnal departure time (minutes after civil dusk) for nine species of songbirds. Age and sex are only included when we could accurately estimate these variables (see Methods)

	Age	Sex	Year
American Redstart (Spring) *Setophaga ruticilla* (*n* = 31)	*F*_1,27_ = 1.2, *P* = 0.283	*F*_1,27_ = 0.6, *P* = 0.456	*F*_1,27_ = 10.6, *P* = 0.003
American Redstart (Spring - two outliers removed) (*n* = 29)	*F*_1,25_ = 0.4, *P* = 0.539	*F*_1,25_ = 1.3, *P* = 0.260	*F*_1,25_ = 0.2, *P* = 0.685
Ovenbird (Spring) *Seiurus aurocapilla* (*n* = 17)	*F*_1,14_ = 0.2, *P* = 0.659	-	*F*_1,14_ = 0.1, *P* = 0.767
Swainson’s Warbler (Spring) *Limnothlypis swainsonii* (*n* = 14)	-	-	F _1,12_ = 1.5, *P* = 0.249
Kirtland’s Warbler (Spring) *Setophaga kirtlandii* *(n* = 66)	*F*_1,62_ = 0.6, *P* = 0.424	*F*_1,62_ = 3.8, *P* = 0.057	*F*_*1*,62_ = 1.1, *P* = 0.307
Kirtland’s Warbler (Fall) *Setophaga kirtlandii* (*n* = 46)	*F*_2,41_ = 0.6, *P* = 0.569	*F*_1,41_ = 2.9, *P* = 0.099	*F*_1,41_ = 0.9, *P* = 0.351
Gambel’s White-crowned Sparrow (Fall) *Zonotrichia leucoPhrys gambelii* (*n* = 49)	-	F_1,46_ = 0.3, P = 0.594	*F*_1,46_ = 0.1, *P* = 0.722
Bicknell’s Thrush (Fall) *Catharus bicknelli* (*n* = 19)	*F*_1,12_ = 0.01, *P* = 0.916	-	*F*_1,12_ = 0.2, *P* = 0.651
Swainson’s Thrush (Fall) *Catharus ustulatus* (Quebec, *n* = 23)	-	-	*Χ*^2^ = 1.8, *df* = 3, *P* = 0.616
Swainson’s Thrush (Fall) *Catharus ustulatus* (Nova Scotia, *n* = 65)	*Χ*^2^ = 2.4, *df* = 1, *P* = 0.124		*Χ*^2^ = 2.1, *df* = 1, *P* = 0.149
Blackpoll Warbler (Fall) *Setophaga striata* *(n* = 49)	*Χ*^2^ = 0.4, *df* = 1, *P* = 0.531	-	*Χ*^2^ = 0.5, *df* = 1, *P* = 0.488
Yellow-rumped Warbler (Fall) *Setophaga coronata* (*n* = 17)	-	-	*Χ*^2^ = 0.04, *df* = 1, *P* = 0.833

**Table 3:** Age, sex, and year differences in sun elevation angle at the time of departure for nine species of songbirds. Age and sex are only included when we could accurately estimate these variables (see Methods)

	Age	Sex	Year
American Redstart (Spring) *Setophaga ruticilla* (*n =* 31)	*F*_1,27_ = 1.6, *P* = 0.214	*F*_1,27_ = 1.0, *P* = 0.327	F_1,27_ = 10.0, *P* = 0.004
American Redstart (Spring - two outliers removed) (*n =* 29)	*F*_1,25_ = 0.4, *P* = 0.556	*F*_1,25_ = 1.3, *P* = 0.270	*F*_1,25_ = 0.2, *P* = 0.660
Ovenbird (Spring) *Seiurus aurocapilla* (*n =* 17)	*F*_1,14_ = 0.2, *P* = 0.670	-	*F*_1,14_ = 0.1, *P* = 0.794
Swainson’s Warbler (Spring) *Limnothlypis swainsonii* (*n =* 14)	-	-	*F*_1,12_ = 1.2, *P* = 0.295
Kirtland’s Warbler (Spring) *Setophaga kirtlandii* (*n =* 66)	*F*_1,62_ = 0.6, *P* = 0.441	*F*_1,62_ = 3.2, *P* = 0.080	*F*_1,62_ = 1.0, *P* = 0.321
Kirtland’s Warbler (Fall) *Setophaga kirtlandii* (*n =* 46)	*F*_2,41_ = 0.4, *P* = 0.685	*F*_1,41_ = 3.8, *P* = 0.057	*F*_1,41_ = 0.8, *P* = 0.373
Gambel’s White-crowned Sparrow (Fall) *Zonotrichia leucophrys gambelii* (*n =* 49)	-	*F*_1,46_ = 1.8, *P* = 0.191	*F*_1,46_ = 0.03, *P* = 0.861
Bicknell’s Thrush (Fall) *Catharus bicknelli* (*n =* 19)	*F*_1,12_ = 0.1, *P* = 0.795	-	*F*_1,12_ = 0.2, *P* = 0.674
Swainson’s Thrush (Fall) *Catharus ustulatus* (*n =* 23)	-	-	*Χ*^2^ = 1.5, *df* = 3, *P* = 0.690
Swainson’s Thrush (Fall) *Catharus ustulatus* (*n = 65*)	*Χ*^2^ = 2.2, *df* = 1, *P* = 0.138		*Χ*^2^ = 2.4, *df* = 1, *P* = 0.125
Blackpoll Warbler (Fall) *Setophaga striata* (*n =* 49)	*Χ*^2^ = 0.2, *df* = 1, *P* = 0.686	-	*Χ*^2^ = 0.01, *df* = 1, *P* = 0.916
Yellow-rumped Warbler (Fall) *Setophaga coronata* (*n =* 17)	-	-	*Χ*^2^ = 0.1, *df* = 1, *P* = 0.752

To determine if the visibility of celestial cues correlated with nocturnal departure time, we compared departure time on overcast (≥ 95% cloud cover) and mostly clear nights (≤ 25% cloud cover), but we found no evidence that cloud cover affected nocturnal departure time. Across all species, 103 of 396 individuals (26%) departed on overcast nights and 148 (37%) departed on mostly clear nights, but we found no significant difference in departure time on overcast nights (41.4 ± 98.3 min after civil dusk) and mostly clear nights (44.8 ± 108.4 min after civil dusk; W = 8591, *n* = 251, *P* = 0.087). Among species likely beginning migration at departure, we found no significant difference in departure time between overcast (33.5 ± 33.6 min after civil dusk) and mostly clear nights (40.3 ± 28.6 min after civil dusk; W = 1613, *n* = 110, *P* = 0.124). Similarly, among species likely first making regional flights, we found no difference in departure times related to cloud cover (overcast = 115.7 ± 199.4 min after civil dusk; mostly clear = 133.8 ± 234.9 min after civil dusk; W = 1069, *n* = 91, *P* = 0.577).

## Discussion

Using automated radio telemetry to monitor departure from breeding and wintering sites by nearly 400 hundred individuals from nine species of songbird, we found that species that were likely embarking on long-distance migration initiated flights with a remarkable degree of synchrony relative to civil dusk. Regardless of species, season, age, or sex, 90% of individuals from species likely initiating migratory flights departed during a 69-minute window beginning at civil dusk. By contrast, species likely initiating non-migratory regional flights departed later and more asynchronously throughout the period between sunset and sunrise. Below, we first explore possible reasons why we detected more synchronous departure than previous studies and then discuss the potential function of early nocturnal departure and its implications for our understanding of avian navigation and orientation.

Despite endogenous control of the diel timing of migration [10–15], the time of nocturnal departure has been hypothesized to be proximately influenced by factors such as age, sex, fuel load, parasite infection, the presence of ecological barriers, and distance remaining to the migratory destination [16, 35]. However, we observed no significant variation in nocturnal departure time by age or sex. Because we studied departure at the onset of migration, there was no variation in the presence of immediate ecological barriers within species, and all individuals had thousands of kilometers remaining on their migratory journey, with the possible exception of Yellow-rumped Warblers breeding in Nova Scotia, whose exact wintering destinations are unknown (see Methods). Furthermore, when comparing departure times between species, those facing a large ocean crossing immediately after departure (i.e., Kirtland’s Warblers, American Redstarts, Ovenbirds, and Swainson’s Warblers departing The Bahamas and Jamaica) did not appear to depart any earlier than species likely beginning migration without an immediate ecological barrier (i.e., Kirtland’s Warblers departing Michigan and Bicknell’s Thrush departing Quebec; Fig. 2). We were unable to investigate other factors that potentially affect nocturnal departure time such as body condition, fuel load, parasite infection, and weather. Weather data were available at our breeding and wintering sites as part of a related study [63], but the hourly weather data were too coarse to assess how weather may have influenced departure time because of the relatively synchronous departure times we observed. Regardless, none of these factors seem likely to account for the large and consistent differences in departure timing observed between species likely beginning migration and those likely first making regional movements (Fig. 2). Instead, we suggest that much of the variation in nocturnal departure time found in our study and in previous studies might be explained largely by unmeasured differences in the types of movements made at departure (i.e., regional or migratory).

Most previous studies have assumed that departure from the study site represents either the onset of migration [6, 7, 9, 64] or its continuation [21, 28, 65]. However, this assumption is not necessarily valid because some birds departing breeding and stopover sites have been shown to initiate regional movements rather than true migratory flights [this study, 31, 39, 42, 43]. Given the diversity of space use strategies observed in winter [6, 66–69], we have no reason to believe that birds departing wintering sites might not also exhibit divergent post-departure movement types, but we did not document regional movements in any of our wintering birds. We suspect that the few late-departing American Redstarts (Fig. 3) may have carried out regional movements after departure, but we had too little tracking data from these specific individuals to assess this intriguing possibility. Clearly, future studies of departure date and time must account for post-departure movement type by tracking individuals after they depart or risk making inappropriate conclusions [31, 40]. Ideally, future researchers will be able to use higher spatial and temporal resolution tracking devices and identify species that show more variation in post-departure movement type within the same population to test our assertions. By doing so, researchers may be able to determine if significant variation in nocturnal departure remains after accounting for post-departure movement type, and if so, what additional factors might be important in explaining this variation.

### Functional significance of nocturnal departure time

Flying at night is thought to be a strategy to reduce energy expenditure and/or reduce predation risk during flight [18, 19], but these benefits could be realized by individuals departing at any time after sunset. Reducing nightly energy expenditure and/or predation risk therefore does not seem to explain why individuals would depart early in the night and with little variation. Instead, we argue that our findings are most consistent with the hypothesis that departing early in the night functions to maximize nightly flight duration [16, 33]. We found that compared to species first making regional movements after departure, which should have no need to maximize nightly flight duration given the short distances travelled, those species that were likely initiating long-distance migratory flights departed earlier and more synchronously relative to civil dusk, thereby maximizing their potential for flight duration and distance. At least anecdotally, this pattern was even evident within a single population, with the four Swainson’s Thrush that likely initiated migratory flights from Nova Scotia departing earlier than individuals first making regional movements.

Somewhat surprisingly, we found little evidence that departure timing varied by age, sex, or season. Migration duration is often shorter in spring than fall [70, 71], and earlier male than female arrival in spring is nearly ubiquitous in birds [72–74]. Nevertheless, the similar departure times we documented in spring and fall and across birds of varying ages and sexes indicates that early nocturnal departure from the breeding and wintering sites is not likely to be part of an individual, seasonal, or sex-specific strategy used to accelerate migration relative to conspecifics [16]. Rather, early nocturnal departure from breeding and wintering sites appears to be part of a more general strategy of departure from the breeding and wintering grounds that birds employ when embarking on long-distance migratory flights, regardless of season, age, or sex.

Holding other important factors constant (i.e., departure date, flight speed, time of flight cessation), individuals departing shortly after civil dusk consistently throughout their migrations could fly longer and farther each night and arrive to their migratory destination days earlier than individuals consistently departing several hours later in the middle of the night. If this were true, early nocturnal departure could be one aspect of a time-minimizing migration strategy that songbirds use to ensure timely arrival to their migratory destinations. However, it is generally not known whether songbirds depart at consistent times during migration. Using some of the only data available to address this question, Liechti et al. [36] found that 90% of Eurasian Hoopoe (*Upupa epops*) and Great Reed Warbler (*Acrocephalus arundinaceus*) flights that lasted more than four hours began shortly after sunset, while flights lasting less than one hour began variably either before sunset or throughout the period between sunset and sunrise. Thus, although there was some variation in the timing of departure across the entire migration period, the vast majority of long-distance migratory flights by Eurasian Hoopoes and Great Reed Warblers began within a narrow range of time after sunset, which suggests that individuals may depart on migratory flights at relatively consistent times throughout migration. However, even if departure times are relatively consistent throughout migration, it is entirely possible that individuals pursuing either time- or energy-minimizing migration strategies depart shortly after civil dusk and instead differ in the frequency of long-distance and short-distance flights, average flight speed, time of flight cessation, and/or time spent at stopover. Only further study of departure times across entire migrations will be able to determine whether early nocturnal departure is part of a time- or energy-minimizing migration strategy, or whether individuals depart shortly after sunset prior to any expected long-distance flight, regardless of their overall migration strategy.

In contrast to birds that immediately begin migration after departure, birds that first make regional flights should be under no pressure to maximize nightly flight duration. By leaving later in the night, Swainson’s Thrush, Blackpoll Warblers, and Yellow-rumpled Warblers breeding in Nova Scotia may be able to reap the energetic and survival benefits of nocturnal flight [18, 19] but also ensure that they arrive at their first post-departure destination during morning twilight when visual habitat cues and social information, such as the presence of conspecifics, would seemingly be easier to detect than in the middle of the night [16, 30]. Exactly how and when migratory birds choose to cease migratory flights, what cues are used to select post-breeding habitat, and the function of these regional movements are all in need of further study [43–45].

### Implications for avian orientation and navigation

To accurately orient and navigate, migratory birds use multiple compasses (i.e., sun, stellar, and geomagnetic) that are calibrated with information derived from celestial cues [22–24], and these cues gradually appear and disappear during evening twilight [20, 21, 59]. Consequently, Åkesson et al. [20, 21] hypothesized that birds should depart within a narrow window of sun elevation angles that corresponds with when the cues most important for orientation and navigation are visible. However, we found that regardless of season, location, or post-departure movement type, individuals departed variably with respect to sun elevation angle, primarily during nautical or astronomical twilight, or later during the night, and this held even among individuals that departed during a narrow window of time. Even within the same species but in different seasons (i.e., Kirtland’s Warbler in spring and fall), we observed no clear preference for departing during a single phase of twilight (Fig. 2), and our results are therefore not consistent with the celestial cues hypothesis as it was originally proposed by Åkesson et al. [20, 21], but are consistent with previous observations of departure timing during migration [20, 21, 30].

Despite the lack of a clear preference for departing during a single phase of twilight, the fact that 98% of individuals in our study waited until after civil dusk to depart, even with extreme differences in the timing of civil dusk relative to sunset (61–109 min after sunset in Alaska vs. 22–31 min after sunset in all other locations), indicates that there is something special about civil dusk. At least two possible explanations exist, and the evidence is equivocal. It could be that the level of darkness present at civil dusk, in addition to the circadian clock, is necessary for full expression of migratory behavior, as has been shown in captive Gambel’s White-crowned Sparrows [15]. Experiments in this species have demonstrated that captive individuals only exhibited all of the behaviors associated with migratory restlessness (i.e., intense locomotor activity, beak-up posture, and beak-up flight) after simulated darkness began, but this requirement of darkness remains untested in other species [15, 75]. Alternatively, if birds need to observe and integrate orientation information prior to departure, but not necessarily at the moment of departure, the availability of celestial cues could set a starting point for how early nocturnal departure can occur without determining the exact time of departure thereafter [30]. At civil dusk, multiple sunset-related orientation cues (i.e., sunset position, horizon glow, polarization patterns) and the brightest stars are all visible [20, 59], which supports the idea that integrating information from multiple cues could be necessary at some point prior to departure. Arguing against this idea, however, is the fact that about a quarter of individuals departed on nights when it was overcast, and departure was not later on overcast nights than on mostly clear nights. When it is overcast during twilight, sunset position may still be discernable, but skylight polarization patterns may no longer be detectable and stars are obscured [60, 76]. However, birds are likely able to integrate information from celestial cues during twilight on the evenings leading up to departure and/or rely solely on geomagnetic information when departing under overcast skies [23, 77]. Ultimately, the role of celestial cues in shaping nocturnal departure time remains unclear and study designs that can differentiate between the potential effects of darkness and the presence of celestial cues are needed.

## Conclusions

Our study reveals the potential power of studying departure from breeding and wintering sites at the beginning of migration. By at least partially controlling for immediate differences in migratory route, proximity to ecological barriers, and distance remaining on migration, we were able to document a long-predicted but seldom observed biological pattern. We observed a remarkable degree of synchrony, relative to civil dusk, in the timing of nocturnal departure by species that were likely initiating migratory flights that was consistent across season, age, and sex. By contrast, we found that species likely carrying out non-migratory regional movements departed later and more asynchronously relative to civil dusk. The fact that nearly all individuals waited until after civil dusk to depart highlights the importance of civil dusk, but it remains unclear if the availability of celestial cues at civil dusk sets a starting point for nightly departure [30] or whether darkness at civil dusk is merely necessary for full expression of migratory behavior [15]. Nonetheless, we argue that early nocturnal departure after civil dusk functions to maximize nightly flight duration and distance and could prove to be a baseline rule of migration. Conceptualizing early nocturnal departure after civil dusk as a baseline rule may allow future studies of departure timing to better understand the social and environmental factors that result in phenological plasticity both at the beginning of and during migration.

## Electronic supplementary material

Below is the link to the electronic supplementary material.

**Additional file 1. Table S1.** Basic descriptive information for populations included in this study. Age classes include hatch year (HY), after hatch-year (AHY), second-year (SY), after second-year (ASY), and unknown (U).

## Data Availability

All detection data from the Motus Wildlife Tracking System (Projects 19, 49, 86, 87, 109, 145) are publicly available at www.motus.org. Weather data were downloaded from the Copernicus Climate Data Store (https://cds.climate.copernicus.eu). All data necessary to reproduce the results are available at https://doi.org/10.25573/data.22505938.
